# Effect of Electroacupuncture with Different Current Intensities on the Serum Metabolomics of Functional Constipation

**DOI:** 10.1155/2023/9693390

**Published:** 2023-07-18

**Authors:** Xiao Wu, Yi Zhou, Guang Chen, CuiHong Zheng, Haoxu Dong, Fan Xiong, Mingmin Zhang, Guangyin Huang, Xiaohu Xu

**Affiliations:** ^1^Department of Integrated Traditional Chinese and Western Medicine, Tongji Hospital, Tongji Medical College, Huazhong University of Science and Technology, 1095 Jiefang Avenue, Wuhan, Hubei 430030, China; ^2^Institute of Integrated Traditional Chinese and Western Medicine, Tongji Hospital, Tongji Medical College, Huazhong University of Science and Technology, 1095 Jiefang Avenue, Wuhan, Hubei 430030, China

## Abstract

**Objective:**

The aim of the study is to investigate the serum metabolomics of electroacupuncture (EA) with different current intensities in the treatment of functional constipation (FC).

**Methods:**

The total number of FC patients was 19, (7, 6, 6, in the low current intensity group (LCI), high current intensity group (HCI), and mosapride citrate tablet control group (MC), respectively). Patients in the EA groups received 16 sessions of acupuncture treatments. Patients in the MC group were orally administered 5 mg mosapride citrate tablets 3 times daily, and serum samples were collected from the patients before and after treatment. Orthogonal partial least square-discriminant analysis (OPLS-DA) was used to assess the metabolic data. The significant differences before and after FC treatment are shown in the OPLS-DA score plot. Variable importance plots (VIPs) and *T* tests were used to identify significant metabolites.

**Results:**

Among the three groups, the number of metabolites with VIP > 1 was 11, 7, and 21 (in LCI, HCI and MC groups, respectively). Compared with those before treatment, the serum metabolites of patients were characterized by increased levels of L-ornithine (*p* < 0.05) and glyceric acid in the LCI group (*p* < 0.05), increased levels of vanillic acid in the MC group (*p* < 0.05), and decreased levels of arabinonic acid in the MC group (*p* < 0.05).

**Conclusions:**

The effects of EA treatment on the serum metabolomics of FC may involve fatty acid and amino acid metabolism.

## 1. Introduction

Functional constipation (FC) is a common functional gastrointestinal disorder (FGID) whose pathophysiological mechanism is not fully understood [1]. Currently, colonic sensorimotor dysfunction [2]; distribution of the interstitial cells of Cajal (ICC) and neuronal cells of the enteric nervous system (ENS) [3, 4]; changes in the intestinal neuroendocrine system [5]; and dysfunction of neurotransmitters, such as substance P, nitric oxide, and acetylcholine, are recognized as important factors affecting gastrointestinal motility.

Mosapride is a selective 5-hydroxytryptamine type 4 (5-HT_4_) receptor agonist and can stimulate gastrointestinal motility [6]. In addition, a study showed that mosapride directly inhibits 5-hydroxytryptamine type 3 (5-HT_3_) receptor functions through a competitive blocking mechanism, likely by binding to the receptor in a closed state, indicating that mosapride has substantial 5-HT_3_ receptor antagonist activity [7]. Thus, mosapride is effective in improving overall symptoms in patients with FGIDs, including chronic gastritis, gastroesophageal reflux diseases, and functional dyspepsia.

Acupuncture has been widely used in the treatment of FGIDs in recent years. Gas chromatography-mass spectrometry (GC-MS) is an analytical technique commonly used in gas chromatography-mass spectrometry and metabolomics, with the advantages of high sensitivity, high accuracy and high separation efficiency, corresponding to the overall regulatory effect of acupuncture, consistent with the integrity and systemic characteristics of traditional Chinese medicine (TCM). In recent years, metabolomics has been extensively applied to explore the mechanism of acupuncture therapy. The feasibility of the application of metabolomics in the study of mechanisms in acupuncture treatment and assessment of therapeutic effects has been confirmed by several studies, and its potential applied value has been conclusively recognized by researchers [8].

Previous studies have shown that FGIDs involve some metabolic disorders to a certain extent, but there is controversy on which related metabolites or pathways are involved. In recent years, metabolomics has also been used to study the mechanism of constipation [9–11]. However, few metabolomics analyses in clinical studies have focused on electroacupuncture (EA) with different current intensities in the treatment of FC. Our previous clinical study demonstrated that EA could significantly improve the defecation frequency, stool properties, and difficulty in defecation of patients with functional constipation [12]. However, its underlying mechanism is still unclear. In this research, GC-MS metabolomics was used to analyze the changes in serum-characterized metabolites in patients with FC before and after EA intervention to identify the differential metabolites closely related to FC and explore the mechanism of electroacupuncture in the regulation of FC.

## 2. Materials and Methods

### 2.1. Reagents and Instruments

Methanol (analytical reagent, Sinopharm Chemical Reagent Co., China), mixed n-alkanes (C7–C40, 49452-U, 1000 *μ*g/mL each component in hexane, Sigma‒Aldrich, USA), bis (trimethylsilyl) trifluoroacetamide-trimethylsilyl chloride (BSTFA-TMCS, 00000000000000033148, BSTFA + TMCS, 99: 1, Sigma‒Aldrich, USA), and triple-distilled water were used.

A GC-MS gas chromatograph-mass spectrometer (Thermo scientific trace 1300GC and signal quadrupole MS, ISQ 1300TM, Thermo, Germany), DB-5MS capillary column (30 m × 0.25 mm × 0.1 *μ*m, Agilent, USA), high-speed cryogenic centrifuge (Eppendorf Research, Germany), vacuum freeze dryer (Virtis Ad Vantage Plus, USA), Microvortex mixer (IKA, Germany), and vacuum centrifuge concentrator (LNG-T88 type, Huamei Biochemical Instrument Factory, Taicang, Jiangsu Province, China) were used.

### 2.2. Patients and Study Design

#### 2.2.1. Patients

Patients with FC who met the Rome III criteria [13] were recruited from December 14, 2011 (first patient enrolled), to March 29, 2015 (last patient completed). The study was performed simultaneously in the following three hospitals in China: Tongji Hospital of Tongji Medical College of Huazhong University of Science and Technology, the Campus Hospital of Huazhong University of Science and Technology, and the Affiliated Hubei Provincial Hospital of Hubei University of TCM.

This study was approved by the Clinical Trial Ethics Committee of Tongji Medical College of Huazhong University of Science and Technology (approval number FWA00007304) and was conducted in accordance with the provisions of the Declaration of Helsinki and good clinical practice guidelines. This study was registered in the Clinical Trials system (ClinicalTrials.gov ID: NCT01274793).

#### 2.2.2. Inclusion Criteria and Exclusion Criteria

We included patients who (1) were aged between 18 and 70 years old; (2) were diagnosed of functional constipation according to the Rome III criteria; (3) did not taking any drugs that promote gastrointestinal motility at least 1 week before randomization; and (4) were willing to sign the informed consent form before randomization. Patients were excluded due to the following conditions: (1) unconsciousness, psychosis, or failure to express subjective symptoms; (2) concomitant serious primary diseases, such as cardiac, hepatic, renal, or hematological disorders; (3) concomitant malignancy or other serious progressive disease; and (4) pregnant or lactating women.

Before enrollment, all patients (1) were asked to stop any medications for FC (e.g., anticholinergic drugs, laxatives, and narcotic), but if the patient did not defecate for more than 3 consecutive days, they could use a glycerin enema (10 ml), and the details were recorded in patient diaries; (2) voluntarily signed the informed consent form with full informed consent.

During screening, each patient underwent laboratory tests, including routine blood, urine, fecal, blood biochemistry (alanine aminotransferase, aspartate aminotransferase, blood urea nitrogen, and serum creatinine) analyses, electrocardiogram and a colonoscopy. Laboratory tests eliminated serious heart, liver, kidney disorders, or other serious illnesses, and colonoscopy excluded organic diseases. For female patients, a urine *β*-human chorionic gonadotropin (HCG) or blood HCG test was required to determine possible pregnancy. All patients received laboratory tests of blood, urine, and feces after completing the treatment. These tests were performed to help assess adverse events.

#### 2.2.3. Interventions

The enrolled patients were randomized into the low current intensity group (LCI), high current intensity group (HCI), and mosapride citrate tablet control group (MC) at a ratio of 1 : 1 : 1. During the 4-week treatment period, patients in the LCI and HCI groups received a total of 16 sessions of acupuncture treatments: 5 times per week for 5 consecutive days in weeks 1 and 2 and 3 times per week on alternate days in weeks 3 and 4. Each session lasted 30 min. In this study, bilateral LI11 (Quchi, located at the midpoint between the lateral end of the transverse cubical crease and the lateral epicondyle of the humerus) and ST37 (Shangjuxu, located on the anterolateral side of the lower leg, the patella and the lateral recess of the patellar ligament 6 inches below, one horizontal finger from the anterior edge of the tibia) were used. LI11 and ST37 are the He-Sea and Lower He-Sea acupoints, respectively, of the large intestine meridians. Both points are considered positive points to treat internal organ disease in TCM and are commonly used for FC.

Sterile disposable acupuncture needles (0.30 × 40 mm or 0.30 × 50 mm, Sino-US Cooperative Taicheng Technology Development Co., Ltd) were used in this study. Then, auxiliary needles (0.18 × 13 mm, Human Health, Shanghai, China) were inserted into the proximal limbs 2 mm lateral to the first needle for 5 mm vertically without manual stimulation. The electroacupuncture instrument (HANS-200E acupoint nerve stimulator, Lianchuang Technology Nanjing Jisheng Medical Technology Co., Ltd.) connects the acupuncture needles and auxiliary needles to form a circuit with a dilatational wave at a frequency of 2/50 Hz. The LCI group was defined as the patients who were administered a low current, which they could clearly perceive, but relatively weakly. The HCI group was defined as the patients who were administered the higher current, which was the maximum current intensity and could be tolerated but did not cause discomfort. Patients in the MC group received oral mosapride citrate (Sumitomo Pharmaceutical Co., Ltd., Japan), 5 mg, 3 times daily, 30 minutes before meals, provided by the Tongji Hospital Pharmacy, Tongji Medical College, Huazhong University of Science and Technology, which was taken continuously for 4 weeks.

#### 2.2.4. Serum Preparation

Serum samples were collected from the 19 FC patients, including 7 in the LCI group, 6 in the HCI group, and 6 in the MC group. Fasting venous blood was collected before and after the interventions. Blood specimens were collected with serum separation gel procoagulation collection tubes, left at room temperature for 30 min, and centrifuged at low temperature (4°C, 3000 r/min, 30 min). The upper layer of serum was stored in the freezer at −80°C.

### 2.3. Sample Processing before Analysis

#### 2.3.1. Extraction

The serum samples were removed from the −80°C refrigerator and thawed at room temperature. Then, 100 *μ*L of the sample was placed into a 2 ml EP tube with 200 *μ*L of methanol and the operation was performed on ice. The samples were centrifuged for 10 min (4°C, 12000 rpm), and 230 *μ*L of supernatant (put into a pointed bottom vapor phase bottle that was washed three times with methanol in advance) was spin dried (4°C, 4000 r, centrifuged for 60 min) and then freeze-dried (overnight for 12 hours). For the blank control group, 100 *μ*L of triple-distilled water was used instead of 100 *μ*L of serum, and the same operation was performed.

#### 2.3.2. Derivate

One hundred microliters of methoxypyridine (15 mg/ml) was added and oxidized in a water bath at 37°C for 120 min. Then, 100 *μ*L of BSTFA containing 1% TMCS was added, silylated in a water bath at 70°C for 30 min, and centrifuged (room temperature, 2000 r, 15 min). Then, the supernatant (150 *μ*L) was added to 50 *μ*L of 50x mixed n-alkanes (C7–C40, 10 *μ*g/mL in hexane) and mixed thoroughly for GC-MS analysis.

#### 2.3.3. GC-MS-Based Metabolomic Analysis

Gas chromatography conditions: a DB-5MS capillary column (30 m × 0.25 mm × 0.1 *μ*m, Gilent, USA) was used with high-purity helium (flow rate 1.0 mL/min) as the carrier gas. The column temperature was ramped up to the initial temperature of 50°C for 10 min and then to 310°C for 20 min at 6°C/min. The injection port temperature was 280°C, the sample was injected without splitting, and the injection volume was 1 *μ*l. The conditions of the mass spectrometer were as follows: ion source temperature 280°C, transmission line temperature 280°C, ionization mode EI, electron energy 70 eV, and acquisition mode: full sweep. The mass-to-nucleus ratio range was 50–650 aum, the scan interval was 0.2 s, and the solvent delay was 5 min.

The samples were alternately rotated between different sets for analysis to reduce the systematic errors caused by instrumental drift.

#### 2.3.4. Quality Control and Verification of GC-MS Methodologies

Before analyzing the samples, the analytical method was first investigated to ensure that accurate and reliable data were obtained. Ten microliters from each sample were mixed together to make a QC sample. Six consecutive injections of the same QC were made to examine the precision of the instrument and ensure that the system was in good condition.

### 2.4. Metabolomics Data Processing and Metabolite Identification

Metabolic data processing and multivariate data analysis: raw format chromatograms were converted to NetCDF format using a Thermo Xcalibur chromatography workstation; raw data signals were extracted using R software (version 2.4.0) retention time correction, peak alignment, deconvolution, and qualitative and quantitative analysis were performed; and the data were analyzed by Simca-P 13.0. OPLS-DA (Orthogonal Partial Least Square-Discriminant Analysis) statistical analysis was performed to obtain score plots, potential differential metabolites were searched for based on VIP > 1, the content difference data were analyzed by SAS 9.2 software (SAS Institute, Cary NC, USA), and statistical analysis (*T* test) was performed. The endogenous metabolites were identified using GC-MS with NIST (National Institute of Standards and Technology) as the mass spectrometry database. The identified differential metabolites were analyzed for metabolic pathways using the HMDB (https://www.hmdb.ca/) and KEGG (Kyoto Encyclopedia of Genes and Genomes, https://www.kegg.jp) databases.

## 3. Results

### 3.1. Patients

The percentages of female patients in the LCI group, HCI group, and MC group were 85.71%, 100%, and 83.33%, respectively. The baseline demographic and clinical characteristics of the patients in the three groups are detailed in Table 1.

### 3.2. Serum Metabolomics

#### 3.2.1. GC-MS Results

**Figure 1:**
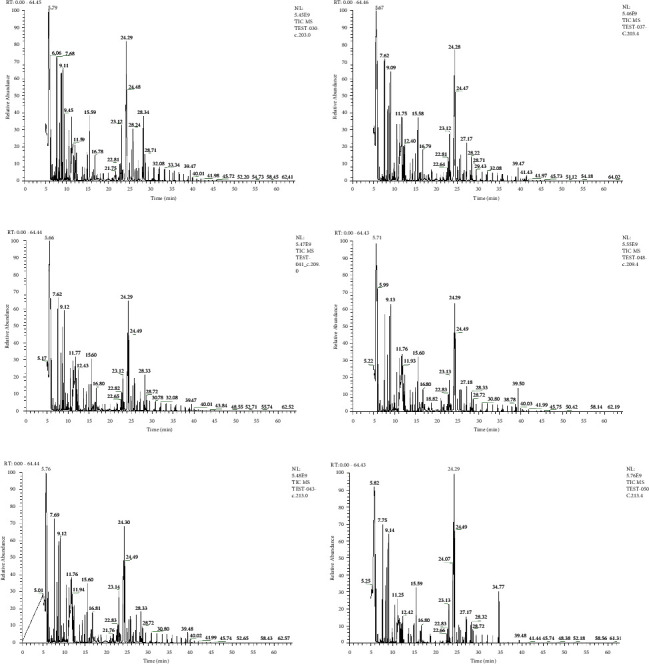
Typical ion flow diagram of patients. (a) Typical ion flow diagram of patients in the LCI group before treatment. (b) Typical ion flow diagram of patients in the LCI group after treatment. (c) Typical ion flow diagram of patients in the HCI group before treatment. (d) Typical ion flow diagram of patients in the HCI group after treatment. (e) Typical ion flow diagram of patients in the MC group before treatment. (f) Typical ion flow diagram of patients in the MC group after treatment.

GC-MS analysis was performed on serum samples from FC patients in the LCI and HCI groups and the MC group before and after treatment, and typical ion flow chromatograms were obtained, as shown in Figure 1. Comparing a, c, and e, there was no significant difference in the ion flow plots between the three groups of patients before treatment, indicating that there was no significant difference in the endogenous metabolic profiles of the three groups of patients before treatment. Comparing b, d, and f, there were some differences in the ion flow plots between the EA groups and the MC group of patients after treatment.

#### 3.2.2. Data Analysis

Orthogonal projections to latent structures discriminant analysis (OPLS-DA), a supervised pattern recognition approach, was used as a predictive model to identify differences in metabolite composition in the samples before and after treatment of FC patients. OPLS-DA can filter out the *X* variables that are unrelated to *Y* and correct the partial least squares project to latent structure (PLS), which can improve the resolution and validity of the model and better distinguish the differences between groups. Model components that are related to *Y* are called predictive, while those that are unrelated to *Y* are called orthogonal.

**Figure 2:**
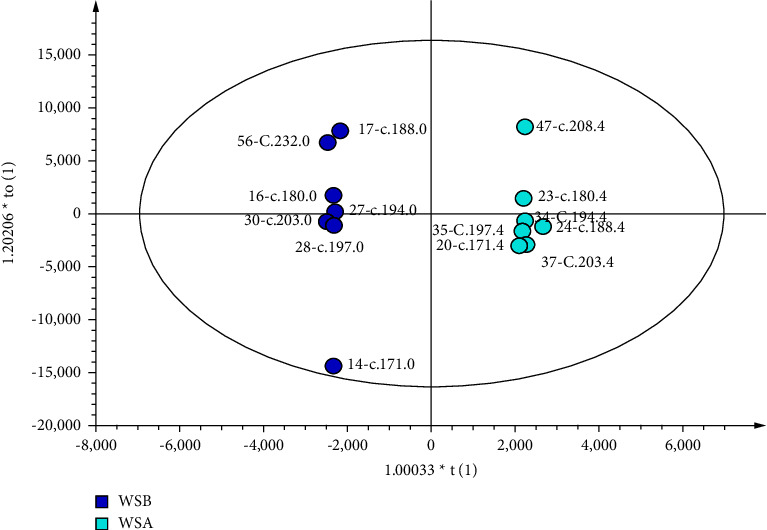
Score plot of the OPLS-DA model of LCI. The left dot represents before treatment (*n* = 7), and the right dot represents after treatment (*n* = 7).

**Figure 3:**
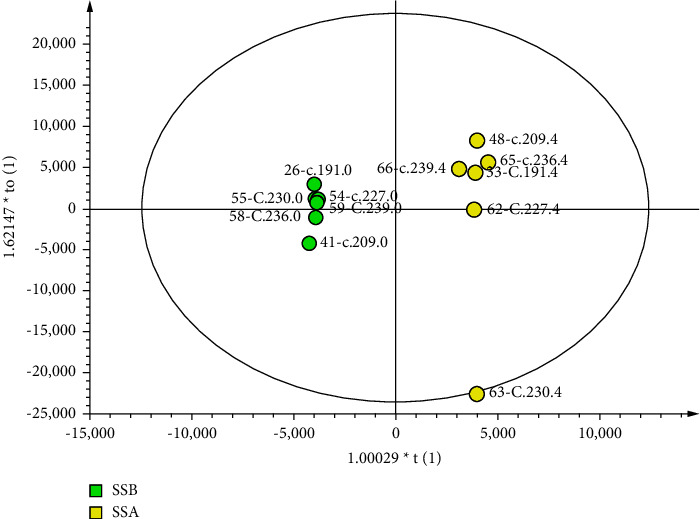
Score plot of the OPLS-DA model of HCI. The left dot represents before treatment (*n* = 6), and the right dot represents after treatment (*n* = 6).

**Figure 4:**
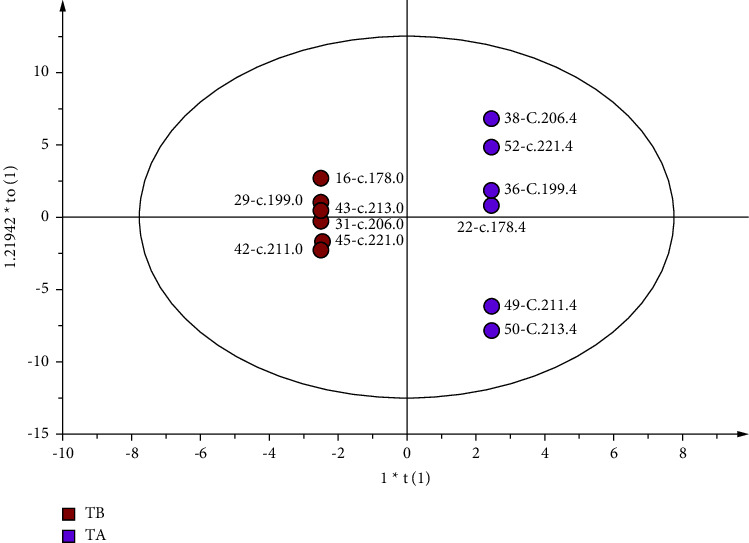
Score plot of the OPLS-DA model of MC. The left dot represents before treatment (*n* = 6), and the right dot represents after treatment (*n* = 6).

The score plots are shown in Figures 2–4. Parameters with a “0” suffix represent pretreatment and parameters with a “4” suffix representing posttreatment. Figures 2–4 show that all parameters (before and after) are distributed within an ellipse (95% confidence interval), with no crossover and complete separation. The results indicate that, after treatment, the serum metabolic phenotypes of patients were significantly changed for both the EA and MC groups.

#### 3.2.3. Quality Control of GC‒MS Methodologies

The quality of the models was determined by the goodness of fit in the *X* (*R*^2^*X*) and *Y* (*R*^2^*Y*) variables. As shown in Table 2, the *R*^2^*X* and *R*^2^*Y* values of the three groups were greater than 0.5, suggesting that the model had stability and reliability, while the *Q*^2^ values of the three groups were greater than 0.99, suggesting that the pretreatment and posttreatment serum samples in the three groups were more predictive and that the metabolite differences were significant.

#### 3.2.4. Identification of Potential Serum Metabolites

Variable importance plots (VIPs) were utilized to select putative metabolites related to acupuncture. The metabolites with VIP > 1 were considered to be compounds that had a greater impact on modeling and contributed more to the separation trend [14]. Among the three groups, the number of metabolites with VIP > 1 was 11, 7, and 21 (in LCI, HCI, and MC, respectively). The variables were identified using the NIST database. The identified metabolites are shown in Table 3.

The statistical analysis indicated that the concentrations of L-ornithine and glyceric acid were increased in the LCI group after treatment (*p* < 0.05, both). In the HCI group, although the VIP values of seven metabolites were greater than 1, none of them were statistically significant after treatment. In the MC group, vanillic acid concentrations decreased after treatment, and arabinonic acid increased (*p* < 0.05, both).

## 4. Discussion

Functional constipation (FC) is a widespread functional gastrointestinal disorder, whose pathogenesis involves diet and physical activity [15]. Diet and physical activity can affect the metabolism. A study showed that fecal metabolites, such as organic acids and esters, could be changed by the interaction of diet and intestinal flora [16].

Acupuncture is an important nonpharmacologic therapy that has been used for managing FC worldwide [17]. EA could improve gastrointestinal motility, promote the contractility of the distal colon, and accelerate whole gut transit in constipated rats [18]. Our previous study demonstrated that EA could significantly improve the defecation frequency, stool properties, and difficulty in defecation of patients with FC [12]. A study indicated that EA could increase the 5-hydroxytryptamine (5-HT) contents of fecal and colon tissues and the expression level of tryptophan hydroxylase (TPH) in colon tissues by stimulating LI11 and ST37 in rats with FC [19]. However, no studies have investigated alterations in metabolomics profiles during the process of electroacupuncture therapy for constipation. We therefore believe that our study provides the first evidence for metabolomics profiles obtained from constipation patients.

In this study, after treatment, glyceric acid and L-ornithine levels increased in the LCI group. Glyceric acid is a novel gastric mucosal protective agent that promotes the synthesis of endogenous prostaglandins in the body [20]. Magierowski et al. [21] found that endogenous prostaglandin E2 (PGE2) largely exists in gastric mucosal cells and has a protective effect on the upper gastrointestinal tract. PGE2 increases blood flow to the gastric mucosa, inhibits gastric acid secretion, and increases the contractile effect of gastrointestinal smooth muscle [22]. Based on this, it can be indirectly inferred that glyceric acid may play a role in the prevention of constipation. L-ornithine is a catalyst and reaction substrate of ornithine carbamoyltransferase and carbamoyl-phosphate synthase and plays an important role in the urea cycle and ammonia detoxification pathway. L-ornithine concentrations were significantly lower in the plasma of patients with colon cancer than those in normal subjects [23]. An animal study indicated that orally administered L-ornithine stimulated gastrointestinal motility, and the stimulatory activity of L-ornithine was mediated by transient receptor potential vanilloid 1 (TRPV1), muscarinic acetylcholine receptor (mAChR), and nitric oxide (NO) synthase [24]. Amino acids can improve tight junction proteins, the function of the intestinal barrier, and the expression of anti-inflammatory cytokines, as well as reduce intestinal cell apoptosis, oxidative stress, and the expression of proinflammatory cytokines in intestinal inflammation [25]. There is substantial evidence linking disturbed gastrointestinal motility to inflammation [26]. Intestinal functions, including motility and secretion, are vulnerable to disruptive influences during inflammatory events. Anti-inflammatory therapy has become a novel target with the potential to improve gastric motor functions [27]. Moreover, the anti-inflammatory effect of acupuncture is a hot topic at present. Therefore, we speculate that the increase in L-ornithine concentration may be related to the mechanism of EA for FC. However, how glyceric acid and L-ornithine mediate protection from constipation remains to be further investigated.

In the MC group, patients had lower vanillic acid concentrations and higher arabinonic acid concentrations than those before treatment. Vanillic acid is a derivative of benzoic acid and is commonly used to make flavor enhancers. Kim et al. [28] found that vanillic acid significantly inhibited the expression of cyclooxygenase-2 (COX-2) and transcription-activating nuclear factor (NF)-*κ*B p65 in the colon tissues of patients with ulcerative colitis induced by sodium dextran sulfate. Furthermore, it reduced the excessive expression level of interleukin 6 (IL-6), which can be used to improve the symptoms of ulcerative colitis. This study identified the role of vanillic acid in the regulation of chronic intestinal inflammation. Arachidonic acid is an oxidation product of arabinose [29]. L-arabinose, one of the isomers of arabinose, can promote intestinal motility and shorten the time to first defecation in constipated mice [30], and it can also alleviate the symptoms of constipation in mice [31]. However, it is unclear whether vanillic acid or arabinonic acid has effects on the symptoms of constipation, which requires further studies. This study may provide a basis for future studies of the metabolic changes in functional constipation.

In a rat model of loperamide-induced constipation, the levels of serum metabolic components, including acetic acid, alanine, glucose, glutamate, glutamine, glycerol, glycine, lactate, succinate, and taurine decreased, with glycine being the most significantly decreased metabolite [32]. After the treatment, glycine concentrations increased significantly [33]. A study indicated that glycine was downregulated in women of reproductive age with chronic constipation; in addition, pathway analysis showed that glycine pathway was significantly altered [34]. Our research found that glycine was present in the metabolic components with VIP > 1 after treatment in all three groups, suggesting that glycine may play an essential role in the progression of constipation. The mechanism is pending further study.

Although the score plots showed a more pronounced trend in metabolite changes in the three groups before and after treatment, few metabolites that were significantly different, similar to the results of previous studies [35, 36]. It was hypothesized that human blood is very complex and highly susceptible to interference by external (environment, temperature, diet, and exercise) or internal factors (chemical reactions among small molecules), and more advanced studies are needed to elucidate the mechanisms involved in these results.

The limitations of this study should be addressed. First of all, because of the cost, only 38 serum samples were collected from 19 patients (7, 6, and 6 in LCI, HCI, and MC, respectively), which might not be sufficiently accurate to reflect the serum metabolomics of EA for FC. This is also why few metabolites showed statistical significance despite many having with VIP > 1. Another limitation is that the fecal metabolite effects of EA on FC were not examined. This study was an extension of a previous clinical study [12]; thus, before the treatment, the fecal samples were not collected from the patients. The results of this study would be more convincing if serum and fecal metabolomics analyses could be combined. In addition, the human metabolism is vulnerable to diet, physical activity, and age. However, first, it is difficult to control the patient's diet and physical activity before collecting blood samples. Second, as noted above, this study was an extension of a previous clinical study; although the inclusion criteria required that the age of participants be from 18 to 70 years old, it is worth noting that the average age of participants in this study was 30 to 40 years old (Table 1). Although we tried to minimize the effect of age on metabolism, the results would be more accurate if there were fewer differences in age. Meanwhile, the 4-week treatment period might not be long enough. Thus, more rigorous and high-quality studies with larger sample sizes are required to prove the metabolomics effects of EA on FC.

## 5. Conclusion

In conclusion, after treatment, serum levels of glyceric acid and L-ornithine increased in the electroacupuncture group, and electroacupuncture for functional constipation may affect fatty acid metabolism and amino acid metabolism in vivo. The mechanism of acupuncture for FC is complex and may involve multiple targets and pathways that are still being explored. This study is meaningful for exploring the serum effects of electroacupuncture on FC from a metabolomics perspective. Due to the limitations of the study sample size, this study cannot yet determine the relationship between metabolomic changes and the efficacy of electroacupuncture in patients with functional constipation, but it lays the foundation for future studies of metabolic changes in constipation disorders.

## Figures and Tables

**Table 1:** Patient demographic and baseline characteristics.

	LCI *n* = 7	HCI *n* = 6	MC *n* = 6	*p*
*Sex, n (%)*				0.63
Female	6 (85.71)	6 (100)	5 (83.33)	
Age (year) mean ± SD	40.57 ± 13.67	34.17 ± 17.56	31.33 ± 12.89	0.52
BMI	21.65 ± 2.68	20.98 ± 1.43	20.92 ± 1.75	0.78
Duration of constipation (months) mean ± SD	183.4 ± 143.79	200.00 ± 232.15	118.00 ± 70.55	0.65

LCI: low current intensity group; HCI: high current intensity group; MC: mosapride control group. BMI: body mass index, kg/m^2^. Continuous values are expressed as the mean (SD), and categorical values are expressed as the *n* (%). *p* values represent the comparison among the three groups.

**Table 2:** Summary parameters for OPLS-DA models before and after treatment.

	Groups	Models	*R* ^2^ *X*	*R* ^2^ *Y*	*Q* ^2^
LCI	Before vs. after	OPLS-DA	0.952	0.541	0.996
HCI	Before vs. after	OPLS-DA	0.951	0.682	0.994
MC	Before vs. after	OPLS-DA	0.850	0.622	1

*R* ^2^ *X* and *R*^2^*Y* show how well the model explains the variation in *X* and *Y*, respectively. *Q*^2^ represents the quality and predictive power of the model: values closer to 1 indicate a more stable and reliable model.

**Table 3:** Serum differential metabolites of the three groups.

	Metabolites	RI	VIP	*p*
LCI	Glycine	1316.23	4.36	↑
	Tyrosine	1892.40	2.69	↑
	L-Ornithine	1763.83	2.45	↑^*∗*^
	Pyroglutamic acid	1530.35	1.80	↑
	Octadecenoic acid	2219.88	1.60	↓
	Glyceric acid	1799.03	1.48	↑^*∗*^
	Octadecenoic acid	2220.48	1.44	↓
	Phenylalanine	1635.85	1.33	↑
	Galactose	1892.94	1.27	↓
	Indole-3-butanoic acid	2193.86	1.14	↑
	Heptadecan-1-ol	2051.66	1.12	↓

HCI	Proline	1309.00	4.42	↑
	Norvaline	1237.46	4.02	↑
	Norleucine	1310.63	2.36	↑
	Octadecenoic acid	2220.48	2.01	↓
	Glycine	1316.23	1.64	↑
	Hexadecanoic acid	2051.56	1.60	↓
	Octadecenoic acid	2219.88	1.19	↓

MC	Vanillic acid	1766.28	1.94	↓^*∗*^
	Arabinonic acid	1763.50	1.92	↑^*∗*^
	Pyridoxal	1824.12	1.77	↓
	Inositol	2074.94	1.76	↓
	*β*-D-fructofuranosyl-(2,1)-beta-D-fructofuranose	2658.71	1.72	↓
	Tagatose	1824.18	1.59	↓
	Malic acid	1562.37	1.58	↓
	Proline	1309.00	1.38	↑
	Lactose	2695.48	1.31	↓
	Dodecanoic acid	1657.82	1.27	↓
	Hexadecanoic acid	2051.56	1.26	↓
	Octadecenoic acid	2219.88	1.23	↓
	Octadecenoic acid	2220.48	1.21	↓
	Glyceric acid	1799.03	1.20	↑
	Docosanoic acid	2644.77	1.17	↓
	Galactitol	1920.76	1.13	↑
	Pyroglutamic acid	1530.35	1.12	↓
	Indole-3-acetaldehyde	1904.03	1.11	↓
	Tocopherol	3006.41	1.11	↓
	Glycine	1316.23	1.04	↑
	Norleucine	1310.63	1.03	↑

^*∗*^ *p* < 0.05 versus before treatment. “↑” represents the concentration of metabolites increased after treatment, whereas “↓” represents the concentration of metabolites decreased after treatment.

## Data Availability

The data that support the findings of this study are available from the first author upon reasonable request.
